# Inhibition of the sonic hedgehog pathway activates TGF-β-activated kinase (TAK1) to induce autophagy and suppress apoptosis in thyroid tumor cells

**DOI:** 10.1038/s41419-021-03744-2

**Published:** 2021-05-08

**Authors:** Sumei Li, Jingxiang Wang, Yurong Lu, Yuqing Zhao, Richard A. Prinz, Xiulong Xu

**Affiliations:** 1 Institute of Comparative Medicine, College of Veterinary Medicine, Yangzhou University, 225009 Yangzhou, Jiangsu Province P. R. China; 2 Department of Surgery, NorthShore University HealthSystem, Evanston, IL USA; 3 Jiangsu Co-innovation Center for Prevention and Control of Important Animal Infectious Diseases and Zoonosis, Yangzhou University, 225009 Yangzhou, Jiangsu Province China; 4 Joint International Research Laboratory of Agriculture and Agri-Product Safety, the Ministry of Education of China, Yangzhou University, 225009 Yangzhou, Jiangsu Province China

**Keywords:** Thyroid cancer, Macroautophagy, Apoptosis, Drug development

## Abstract

The sonic hedgehog (Shh) pathway is highly activated in a variety of malignancies and plays important roles in tumorigenesis, tumor growth, drug resistance, and metastasis. Our recent study showed that the inhibitors of the Shh pathway such as cyclopamine (CP), a Smothened (SMO) inhibitor, and GANT61, a Gli1 inhibitor, have modest inhibitory effects on thyroid tumor cell proliferation and tumor growth. The objective of this study was to determine whether autophagy was induced by inhibition of the Shh pathway and could negatively regulate GANT61-induced apoptosis. Here we report that inhibition of the Shh pathway by Gli1 siRNA or by cyclopamine and GANT61 induced autophagy in SW1736 and KAT-18 cells, two anaplastic thyroid cancer cell lines; whereas Gli1 overexpression suppressed autophagy. Mechanistic investigation revealed that inhibition of the Shh pathway activated TAK1 and its two downstream kinases, the c-Jun-terminal kinase (JNK) and AMP-activated protein kinase (AMPK). GANT61-induced autophagy was blocked by TAK1 siRNA and the inhibitors of TAK1 (5Z-7-oxozeaenol, 5Z), JNK (SP600125), and AMPK (Compound C, CC). Inhibition of autophagy by chloroquine and 5Z and by TAK1 and Beclin-1 siRNA enhanced GANT61-induced apoptosis and its antiproliferative activity. Our study has shown that inhibition of the Shh pathway induces autophagy by activating TAK1, whereas autophagy in turn suppresses GANT61-induced apoptosis. We have uncovered a previously unrecognized role of TAK1 in Shh pathway inhibition-induced autophagy and apoptosis.

## Introduction

Thyroid cancer is one of the most common endocrine malignancies^1^. Thyroid cancers are divided into several different tumor types based on the pathological characteristics and the origins of tumor cells^2^. Well differentiated papillary (PTCs) and follicular (FTCs) thyroid cancers are derived from thyroid follicular epithelial cells and may progress into poorly differentiated or anaplastic thyroid carcinomas (ATCs)^1^. Medullary thyroid cancers (MTCs) are derived from the C cells of the thyroid gland. Surgery, thyroid hormone therapy, and radioiodine can cure most differentiated PTCs and FTCs but are not effective for poorly differentiated thyroid cancer^3^. Approximately 15–20% of thyroid cancer patients develop recurrence in their lifetime^1^. Currently, there is no effective therapy to treat ATC. The mean survival of ATC patients is only 2–6 months^4^.

The Shh pathway has been implicated in a plethora of tumor promoting activities including cell proliferation, epithelial to mesenchymal transition (EMT), angiogenesis, drug resistance, and metastasis^5^. The Shh pathway is activated when one of the hedgehog ligands (Sonic, Indian, or Desert) engages with their shared Patched (Ptch) receptor, a 12-pass transmembrane protein. Smoothened (Smo), a G-protein-coupled seven-pass transmembrane protein, then dissociates from Ptch and is translocated into the primary cilia where it becomes activated^6,7^. Activated Smo disrupts the interaction between Sufu and Gli1, a latent zinc-finger transcription factor that regulates the expression of many genes involved in cell proliferation, apoptosis, stem cell self-renewal, and drug resistance. The *PTCH* and *SMO* genes are frequently mutated in basal cell carcinomas and neuroblastomas. The Shh pathway is also highly activated in various tumor types ranging from extremely malignant pancreatic and lung cancers to the relatively benign well differentiated thyroid cancer. The Shh pathway can be cross-activated by the phosphatidylinositol-3 (PI)-3 and mitogen-activated protein (MAP) kinase pathways, and through paracrine activation by Shh-secreting tumor stromal cells^7,8^. Targeting the Shh pathway is a potential novel therapeutic strategy to treat cancers^6^. Indeed, several Smo inhibitors have been approved for treating basal cell carcinomas (BCCs) and neuroblastomas but these inhibitors have little success in cancers that lack the *PTCH* or *SMO* gene mutation^6^. Our recent study showed that GANT61, a Gli1 inhibitor, is able to control the growth of thyroid cancer stem cell-derived tumor but not effective to control the growth of bulk cell-derived tumors in a xenograft mouse model^9^. Understanding the molecular mechanisms for the poor performance of Gli1 inhibitors is imperative to improve the anticancer activity of Shh pathway inhibitors.

Autophagy is an evolutionarily conserved self-digestion process used by eukaryotic cells for energy recycling and for eliminating misfolded proteins and damaged organelles. Autophagy starts with the expansion of the crescent endosomal membrane that then engulfs the neighboring cargos in the cytosol to form a double-membraned vesicle, the autophagosome^10^. This process is triggered when cells sense various forms of stress such as nutrient and energy starvation, protein aggregates, and intracellular microbes^10^. Biochemically, nutrient and energy deprivation leads to ULK1 activation by the mammalian target of rapamycin (mTOR) inaction and AMPK activation, respectively. mTOR phosphorylates UNC-51-like kinase 1 (ULK1)^S757^ and inhibits ULK1 activity, whereas AMPK phosphorylates ULK1^S555/777/317^ and activates ULK1 (ref. ^10^). ULK1 activates a cascade of signaling molecules involved in the formation of the pre-initiation complex and the autophagosomes^10^. Mature autophagosomes fuse with lysosomes to form autolysosomes in which engulfed contents are degraded by proteases.

Autophagy has been implicated in playing an important role in antagonizing the therapeutic effects of various anticancer drugs. Several studies have shown that inhibition of the Shh pathway can induce autophagy. For example, GANT61, a Gli1 inhibitor, induces autophagy, whereas Shh suppresses autophagy in hepatocellular carcinoma cells^11^. GANT61 induces autophagy in *MCYN*-amplified neuroblastoma cell lines^12,13^. Vismodegib, a Smo inhibitor, induces autophagy in human lung cancer cells^14^. However, the mechanisms of Shh pathway inhibition-induced autophagy remain largely unknown. Whether autophagy represses or enhances the Shh pathway inhibition-mediated anticancer activity remains controversial. Our present study focuses on the mechanisms of Shh pathway inhibition-induced autophagy in thyroid cancer cells and its role in apoptosis. Here we report that inhibition of the Shh pathway induced autophagy by activating TAK1, a MAP kinase kinase kinase (MAP3K) that then activated two downstream kinases, JNK and AMPK. Inhibition of autophagy enhanced GANT61-induced apoptosis in two thyroid tumor cell lines. Our study uncovers a previously unrecognized role of TAK1 in mediating Shh pathway inhibition-induced autophagy and apoptosis.

## Results

### Inhibition of the Shh pathway induces autophagy

**Fig. 1:**
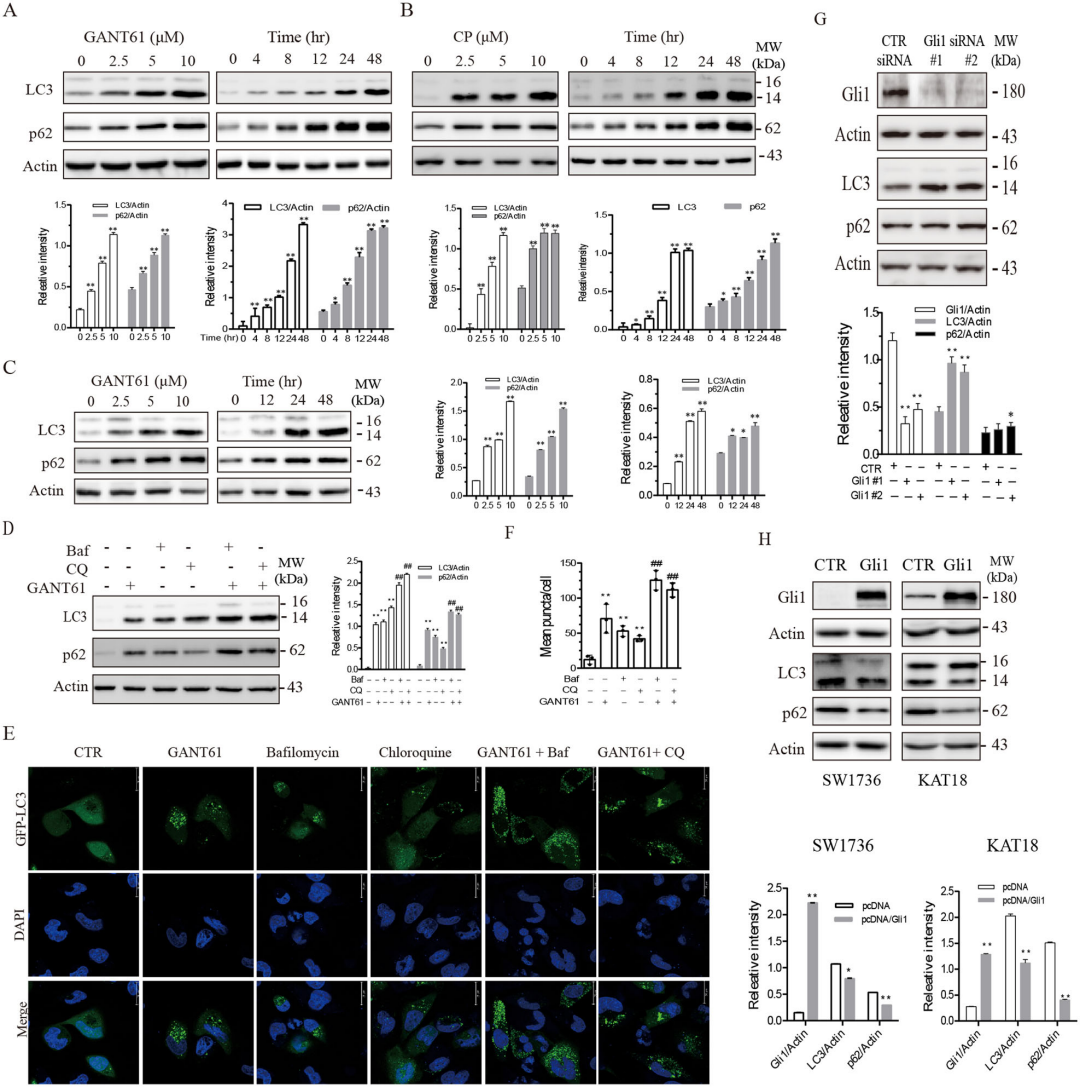
Inhibition of the Shh pathway induces autophagy. SW1736 (**A**) and KAT-18 (**C**) cells were treated with the indicated concentrations of GANT61 for 48 h or with 10 μM GANT61 for the indicated lengths of time. **B** SW1736 cells were treated with the indicated concentrations of cyclopamine (CP) for 48 h or with 10 μM CP for the indicated lengths of time. LC3, p62, and β-actin were analyzed by western blot. **D** The effect of bafilomycin and chloroquine on GANT61-induced autophagy. SW1736 cells were incubated in the absence or presence of GANT61 (10 μM) for 24 h. Bafilomycin (Baf) (10 nM) or chloroquine (CQ) (10 μM) was added and incubated for another 8 h. LC3 lipidation, p62, and actin levels were analyzed by western blot. Due to the low level of LC3 I, the ratio of LC3-II to β-actin was used for autophagy analysis in this and the remaining figures. Protein band density was analyzed by using NIH Image-J software. Data presented as the mean ± SD (*n* = 3) relative to control are shown in bar graphs. *p < 0.05, **p < 0.01, compared to untreated control; ^##^*p* < 0.01, compared to GANT61 (**A**–**D**). **E**, **F** GANT61 induces autolysosome formation. SW1736 cells transiently transfected with an expression vector encoding the GFP-LC3 gene were left untreated or treated with GANT61 (10 μM) for 24 h with or without bafilomycin (10 nM) or CQ (10 μM). After nuclear staining with DAPI, green puncta were visualized under a confocal microscope and statistically analyzed. Bar length: 20 μm. **p < 0.01, compared to untreated controls; ^##^*p* < 0.01, compared to GANT61. **G** SW1736 cells were transfected with control or Gli1 siRNA. After incubation for 48 hr, the cells were harvested and analyzed for Gli1, p62, LC3, and β-Actin by western blot. **H** SW1736 and KAT-18 cells were transfected with an empty vector or the vector encoding the *GLI1* gene. After incubation for 48 h, the cells were harvested and analyzed for Gli1, p62, LC3, and β-Actin by western blot. Relative protein levels were analyzed by quantification of the density of the protein bands with NIH Image-J software and presented as bar graphs. Data are the mean ± SD of three experiments. *p < 0.05, **p < 0.01, compared to the transfection control (**E**, **F**).

GANT61, a Gli1-specific inhibitor, induced LC3 lipidation in two anaplastic thyroid cancer cell lines, SW1736 (Fig. 1A) and KAT-18 cells (Fig. 1C), in a dose- and time-dependent manner. Consistently, cyclopamine (CP), an inhibitor of Smo, also increased microtubule-associated proteins 1A/1B light chain (LC3) lipidation in a dose- and time-dependent manner in SW1736 cells (Fig. 1B). p62 is a ubiquitin-binding protein that sequesters ubiquitinated proteins for lysosomal degradation through interacting with LC3-II in autophagosomes^15^. p62 levels are usually decreased in cells undergoing autophagy. Here we found that both GANT61 and CP increased p62 levels in a time- and dose-dependent manner in SW1736 and KAT-18 cells (Fig. 1A–C). To investigate if increased LC3 lipidation was due to the induction of autophagy or due to the stall of the autophagy flux, we investigated the effect of bafilomycin (Baf) and chloroquine (CQ) on GANT61-induced autophagy. As shown in Fig. 1D, GANT61 (10 μM), bafilomycin (10 nM), and CQ (10 μM) alone increased LC3 lipidation and p62 levels. The combination of GANT61 with bafilomycin or CQ further increased LC3 lipidation and p62 levels compared to bafilomycin or CQ alone. Confocal microscopy revealed that GANT61 (10 μM), bafilomycin (10 nM), and CQ (10 μM) all induced the formation of green fluoresce protein (GFP)-tagged LC3 puncta perinuclearly distributed in SW1736 cells (Fig. 1E). Enumeration of autophagosomes revealed that GANT61, bafilomycin, and CQ significantly increased the number of GFP-LC3 puncta (Fig. 1F). GANT61 in combination with bafilomycin or CQ further increased the number of GFP-LC3 puncta, compared to those treated with bafilomycin or CQ alone (Fig. 1E, F). These results collectively suggest that GANT61 induces autophagy, and that increased LC3-II levels is not due to the inhibition of the autophagy flux.

### Gli1 regulates autophagy

Gli1 is a transcription factor that plays a central role in executing many biological functions of the Shh pathway^7,8^. Here we investigated the role of Gli1 in mediating GANT61-induced autophagy. We first examined the effect of Gli1 knockdown on autophagy in SW1736 cells. As shown in Fig. 1G, Gli1 expression was very effectively suppressed in SW1736 cells transfected with two Gli1 siRNAs, compared to that transfected with the control siRNA. Gli1 knockdown led to increased LC3 lipidation and p62 levels. In contrast, Gli1 overexpression in SW1736 and KAT-18 cells significantly lowered LC3 lipidation and p62 levels (Fig. 1H).

### Inhibition of the Shh pathway leads to TAK1 activation

**Fig. 2:**
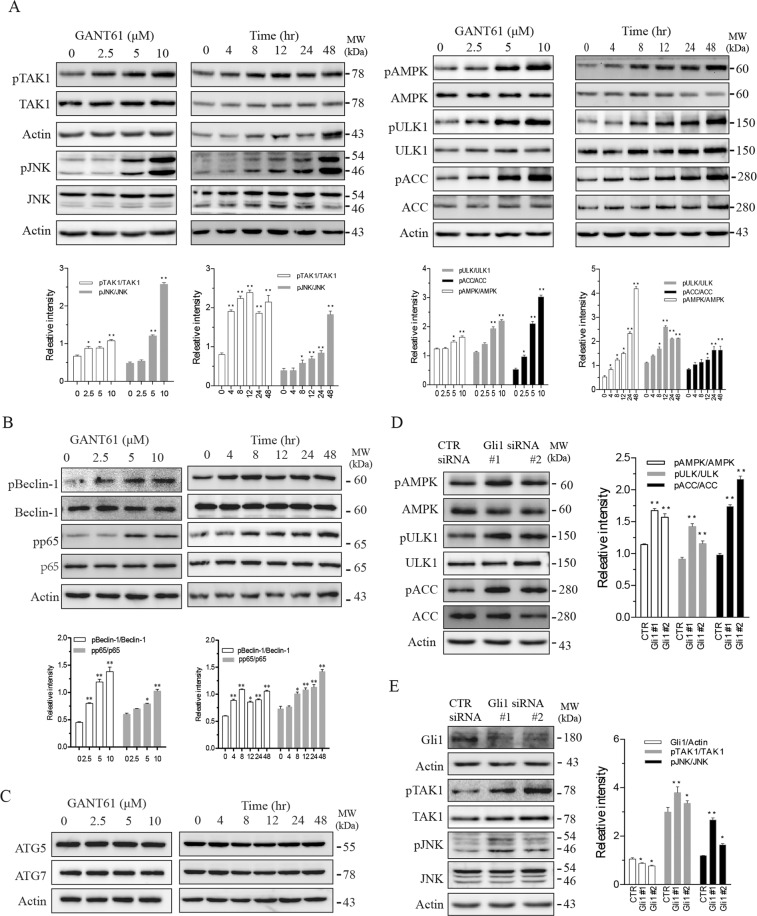
Gli1 inhibition activates TAK1 and its downstream kinases. **A**–**C** SW1736 cells were treated with the indicated concentrations of GANT61 for 48 h or with 10 μM GANT61 for the indicated lengths of time. **D**, **E** SW1736 cells were transfected with control or Gli1 siRNA and incubated for 48 h. Cell lysates were analyzed for protein phosphorylation and their total protein by western blot with the indicated antibodies. Protein band density was analyzed by using an NIH Image-J software. Data presented as the mean ± SD (*n* = 3) relative to control are shown in bar graphs. *p < 0.05, **p < 0.01, compared to the untreated control or the scrambled siRNA control.

It has been increasingly recognized that TAK1, a serine/threonine kinase activated by various stimuli, plays an important role in autophagy and apoptosis^16^. TAK1 phosphorylates JNK and AMPK, both of which are involved in autophagy^17^. Here we tested if suppression of the Shh pathway led to TAK1 activation. As shown in Fig. 2A, GANT61 induced TAK1^S412^ phosphorylation in SW1736 cells in a time- and dose-dependent manner. GANT61 also increased phosphorylation of several downstream kinases, including JNK^T183/185^ and AMPK^T172^ as well as ULK1 (Unc-52-like kinase 1)^S555^ and ACC (acetyl-CoA carboxylase)^S79^, two substrates phosphorylated by AMPK. In addition to JNK, TAK1 phosphorylates and activates several other downstream molecules including NF-κB, p38, and ERK^18^. As shown in Fig. 2B, GANT61 increased p65^S539^ phosphorylation of NK-κB. Beclin-1 is an autophagy -related molecule downstream of AMPK^10^. Beclin-1 phosphorylation leads its dissociation from the Bcl-2-Beclin complex^10^. GANT61 dose- and time-dependently increased Beclin-1^S93^ phosphorylation (Fig. 2B). GANT61 did not affect the levels of Beclin-1 and other two autophagy-regulated proteins, ATG5 and ATG7 (Fig. 2B, C). Consistent with these observations, Gli1 suppression by two siRNAs also led to increased phosphorylation of JNK^T183/185^, AMPK^T172^, ULK1^S555^, and ACC^S79^ (Fig. 2D, E).

### TAK1 is required for GANT61-induced autophagy

**Fig. 3:**
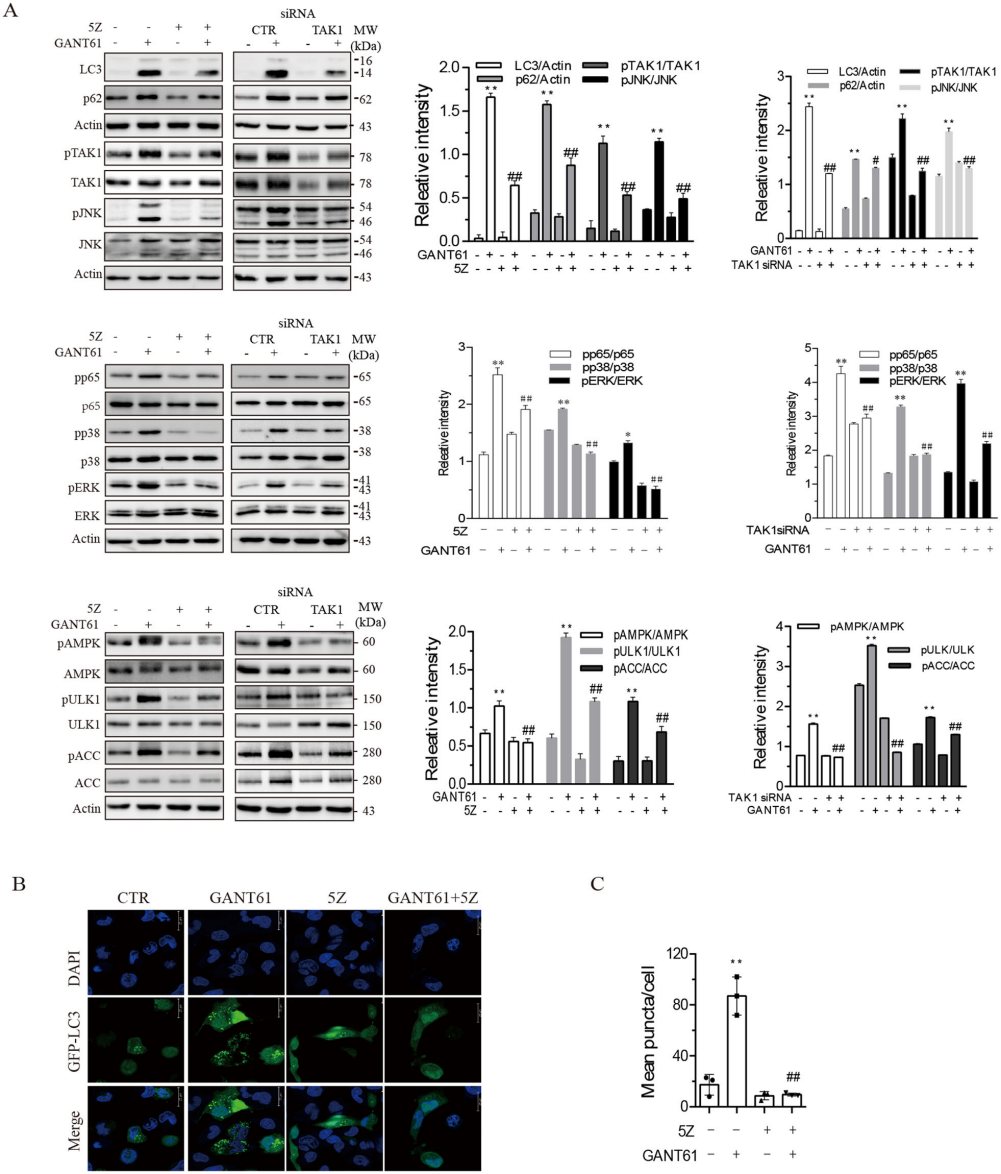
TAK1 mediates GANT61-induced autophagy. **A** SW1736 cells were incubated in the absence or presence of GANT61 (10 μM) minus or plus 5Z (1 μM) for 48 h. Alternatively, SW1736 cells were transfected with scrambled or TAK1 siRNA (2.5 nmole each). After incubation for 4 h, the cells were left untreated or treated with GANT61 (10 μM) for 48 h. Cell lysates were prepared and analyzed for total and phosphorylated proteins by western blot. The expression levels were analyzed by quantification of the density of the protein bands with NIH Image-J software and presented as bar graphs. *p < 0.05; **p < 0.01, compared to the untreated control; ^#^*p* < 0.05; ^##^*p* < 0.01, compared to GANT61-treated SW1736 cells. **B**, **C** Inhibition of GANT61-induced autolysosome formation by 5Z. SW1736 cells transiently transfected with the expression vector encoding the GFP-LC3 gene were incubated in the absence or presence of GANT61 (10 μM) minus or plus 5Z (1 μM) for 48 h. After nuclear staining with DAPI, the cells were examined under a confocal microscope for the presence of autophagosomes (**B**). The number of autophagosome puncta per cells was statistically analyzed (**C**). Bar length: 20 μm. **p < 0.01, compared to untreated control; *##p* < 0.01, compared to GANT61.

We next determined if inhibition of TAK1 by 5Z-7-oxozeaenol (5Z), a TAK1-specific inhibitor, could block GANT61-induced autophagy. As shown in Fig. 3A, 5Z blocked GANT61-induced LC3 lipidation and p62 expression. 5Z also blocked GANT61-induced phosphorylation of TAK1^S412^, JNK^T183/185^, p65^S93^, p38^T180/182^, ERK^T202/204^, AMPK^T172^, ULK1^S555^, and ACC^S79^. TAK1 siRNA very effectively suppressed TAK1 expression as well as TAK1^S412^ phosphorylation (Fig. 3A). TAK1 knockdown had similar effects on GANT61-induced protein phosphorylation and LC3 lipidation (Fig. 3A). Consistently, 5Z blocked GANT61-induced formation of autophagosomes in SW1736 cells (Fig. 3B, C).

### AMPK and JNK are required for GANT61-induced autophagy

**Fig. 4:**
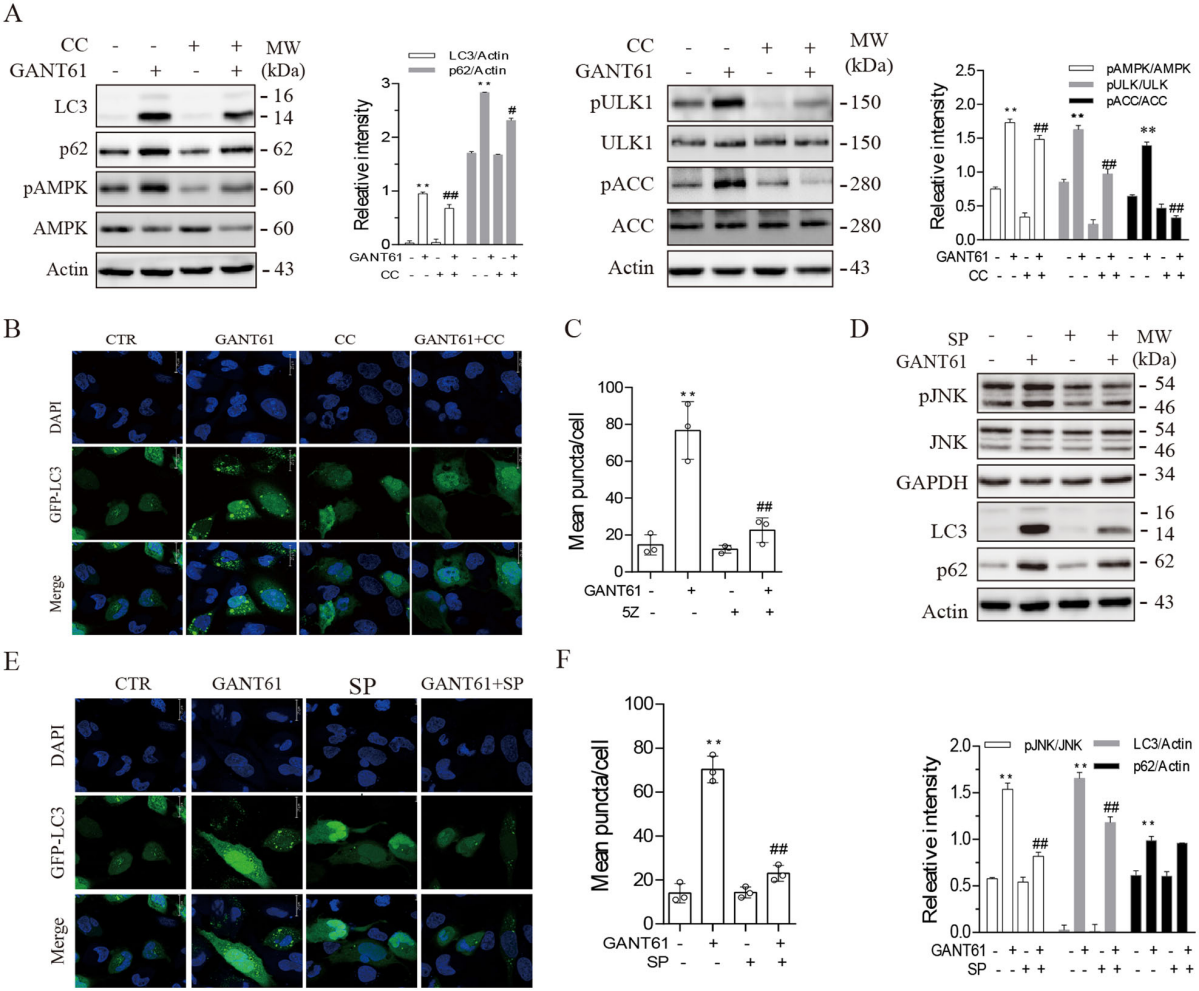
AMPK and JNK are required for GANT61-induced autophagy. SW1736 cells were incubated in the absence or presence of GANT61 (10 μM) minus or plus compound C (5 μM) (**A**) or SP600125 (10 μM) (**D**) for 48 h. Cell lysates were prepared and analyzed for total and phosphorylated proteins by western blot. The expression levels were analyzed by quantification of the density of the protein bands with NIH Image-J software and presented as bar graphs. **B**, **C**, **E**, **F** SW1736 cells transiently transfected with the expression vector encoding the GFP-LC3 gene were incubated in the absence or presence of GANT61 (10 μM) minus or plus compound C (5 μM) (**B**, **C**) or SP600125 (10 μM) (**E**, **F**) for 48 h. After nuclear staining with DAPI, the cells were examined under a confocal microscope for the presence of autophagosomes (**B**, **E**). The number of autophagosome puncta per cells was statistically analyzed (**C**, **F**). Bar length: 20 μm. **p < 0.01, compared to untreated control; ^*##*^*p* < 0.01, compared to GANT61.

AMPK phosphorylates ULK1^S555^ and activates its enzymatic activity to trigger autophagy^19,20^. Here we tested if GANT61-induced autophagy was indeed mediated through TAK1-activated AMPK. As shown in Fig. 4A, compound C (CC), an inhibitor of AMPK, blocked GANT61-induced ULK1^S555^ phosphorylation and LC3 lipidation. CC inhibited AMPK^T172^ and ULK1^S555^ phosphorylation in GANT61-treated SW1736 cells. Consistently, CC blocked GANT61-induced formation of autophagosomes in SW1736 cells (Fig. 4B, C). JNK phosphorylates Bcl-2 and induces its dissociation with Beclin-1 (Klionsky et al.^10^). We then tested whether inhibition of JNK also blocked GANT61-induced autophagy. As shown in Fig. 4D, SP600125, a specific inhibitor of JNK, blocked GANT61-induced JNK^T183/185^ phosphorylation and LC3 lipidation. Consistently, SP600126 blocked GANT61-induced formation of autophagosomes in SW1736 cells (Fig. 4E, F).

### Autophagy inhibition enhances GANT61-induced apoptosis

**Fig. 5:**
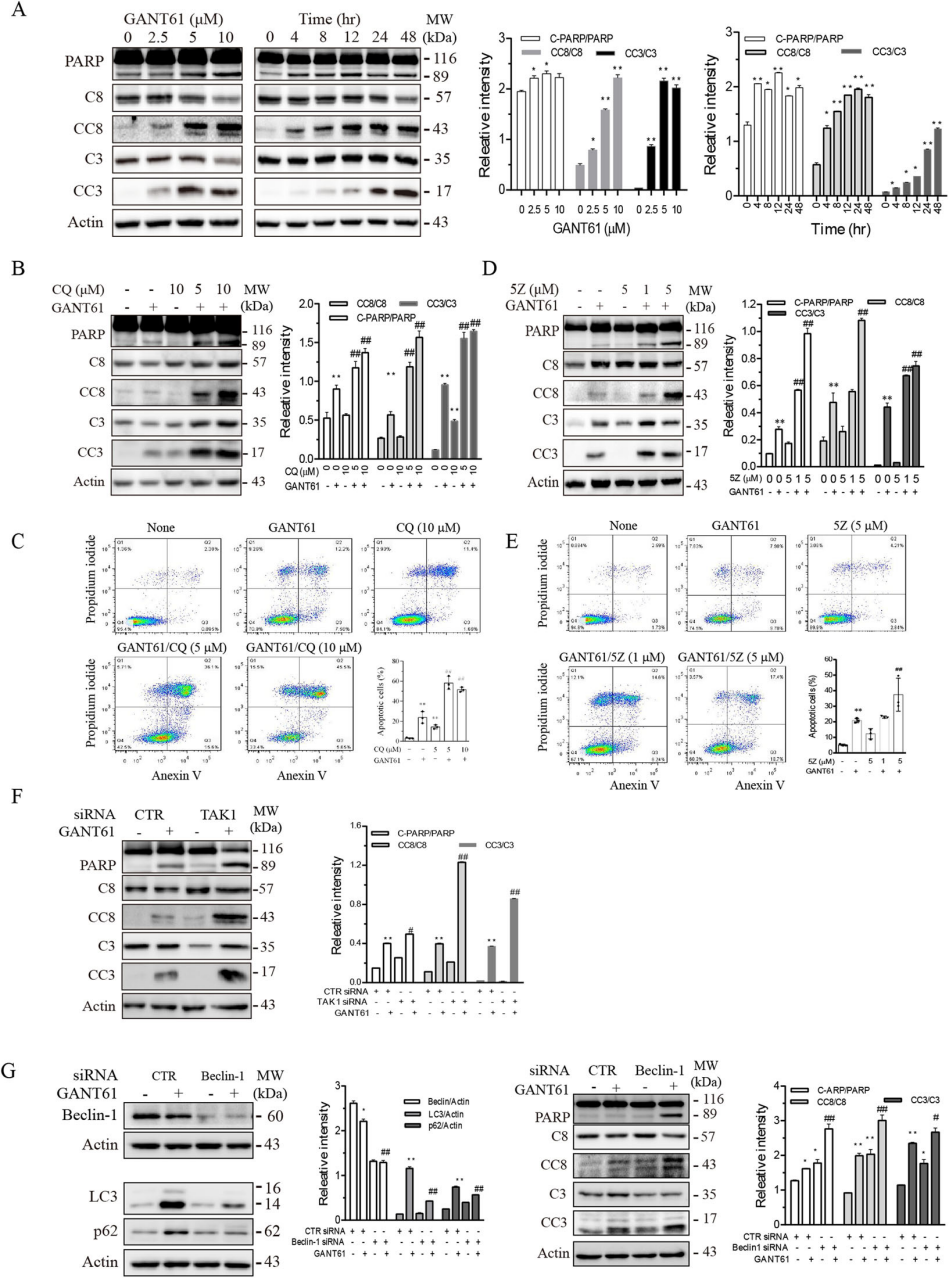
Inhibition of autophagy enhances GANT61-induced apoptosis. **A** SW1736 cells were treated with the indicated concentrations of GANT61 for 48 h or with 10 μM GANT61 for the indicated lengths of time. **B**, **D** SW1736 cells were incubated in the absence or presence of GANT61 (10 μM) minus or plus 5Z (1 or 5 μM) (**B**) or CQ (5 or 10 μM) (**D**) for 24 h. Cell lysates were prepared and analyzed for PARP, caspase-8 (C8), cleaved caspase-8 (CC8), caspase-3 (C3), and cleaved caspase-3 (CC3) by western blot. C-PARP cleaved PARP. **C**, **E** SW1736 cells were incubated in the absence or presence of GANT61 (10 μM) minus or plus 5Z (1 or 5 μM) (**C**) or CQ (5 or 10 μM) (**E**) for 24 h. Single-cell suspensions were stained for propidium iodide (PI) and annexin V followed by flow cytometry. Data in bar graphs are the mean ± SD of three independent experiments. **F**, **G** SW1736 cells were transfected with scrambled or TAK1 siRNA (**F**) or Beclin-1 siRNA (2.5 nmole each) (**G**). After incubation for 24 h, the cells were left untreated or treated with GANT61 for 24 h. Cell lysates were analyzed for p62, LC3, Beclin-1, TAK1, PARP, C8, CC8, C3, CC3, and β-Actin by western blot. The expression levels were analyzed by quantification of the density of the protein bands with NIH Image-J software and presented as bar graphs. *p < 0.05; **p < 0.01, compared to the untreated control; ^#^*p* < 0.05; ^*##*^*p* < 0.01, compared to GANT61 (**A**–**E**).

It is well documented that autophagy antagonizes the antitumor effect of various anticancer drugs in part by suppressing apoptosis^21^. Here we investigated whether GANT61-induced autophagy also impacted GANT61-induced apoptosis. As shown in Fig. 5A, GANT61 induced caspase-3 (C3) and caspase-8 (C8) in a dose- and time-dependent manner but induced poly (ADP-ribose) polymerase (PARP) cleavage largely independent of the dose response. Notably, GANT61 activated caspase-8 and PARP slightly earlier than caspase-3 (Fig. 5A). Inhibition of autophagy by chloroquine (5 or 10 μM; Fig. 5B) and by inhibiting TAK1 activity with 5Z (1 or 5 μM; Fig. 5D) and by TAK1 siRNA (2.5 nmole; Fig. 5F) enhanced GANT61-induced caspase-8 and PARP cleavage more potently than caspase-3. To further confirm the effect of autophagy on GANT61-induced apoptosis, we conducted flow cytometry to analyze the expression of annexin V on the cell surface. As shown in Fig. 5C, E, chloroquine (5 or 10 μM) or 5Z (5 μM) in combination with GANT61 (10 μM) significantly increased the percentage of annexin V-positive cells, compared to those treated with GANT61, chloroquine, or 5Z alone. Finally, we investigated if inhibition of autophagy by Beclin-1 knockdown also potentiated GANT61-induced apoptosis. As shown in Fig. 5G, Beclin-1 siRNA significantly silenced Beclin-1 expression in SW1736 cells. Beclin-1 knockdown blocked GANT61-induced LC3 lipidation and p62 expression. Consistently, Beclin-1 silencing also enhanced GANT61-induced caspase-8, caspase-3, and PARP cleavage (Fig. 5G). These observations collectively suggest that inhibition of autophagy potentiates GANT61-induced apoptosis.

### Autophagy inhibition enhances GANT61-induced antiproliferative effects

**Fig. 6:**
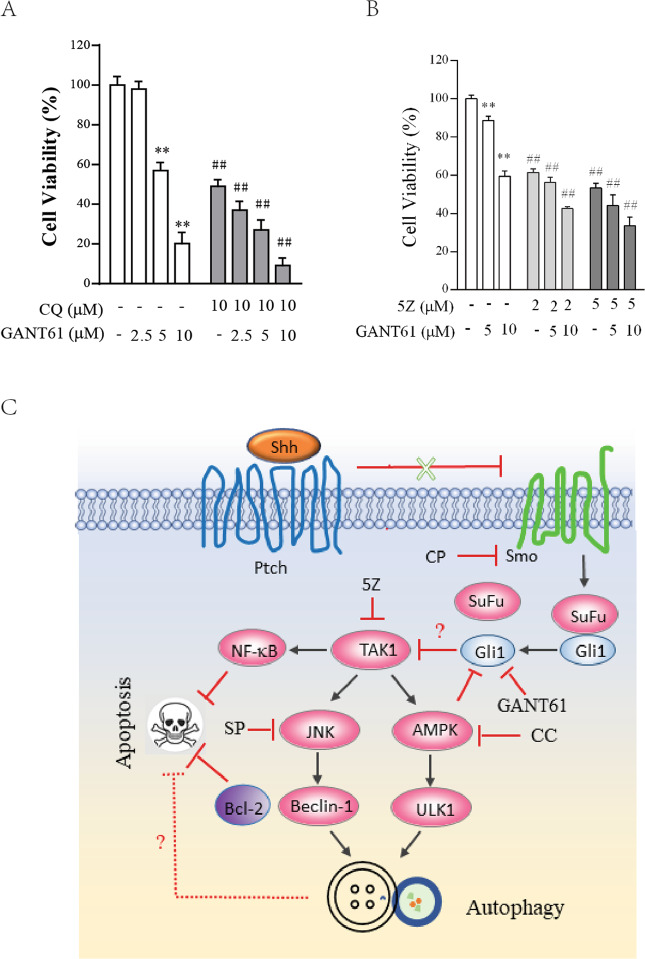
Inhibition of autophagy potentiates the antiproliferative effect of GANT61. SW1736 cells seeded in 96-well plates (2000 cells/well) were incubated with the indicated concentrations of GANT61 minus or plus CQ (10 μM) (**A**) for 72 h or 5Z (2 or 5 μM) (**B**) for 48 h. Cell viability was measured by using a CellTiter-Glo kit. Data are the mean ± SD of the triplicate from one representative of three independent experiments with similar results. **p < 0.01, compared to the untreated control; ^*##*^*p* < 0.01, compared to the counterparts in SW1736 left untreated or treated with the same concentrations of GANT61. **C** The mechanisms of Shh pathway inhibition-induced autophagy. Shh binding to Ptch releases the restriction of Smo activity. Activated Smo disrupts the interaction between Sufu and Gli1. Inhibition of the Shh pathway by the Smo inhibitor cyclopamine or by the Gli1 inhibitor GANT61 activates TAK1, which then activates JNK and AMPK. JNK promotes autophagy by phosphorylating Bcl-2 and dissociating it from Beclin-1. Free Beclin-1 becomes available for the initiation of autophagy. AMPK phosphorylates ULK1 and activates it. ULK1 is involved in the assembly of the pre-initiation complex and the early formation of autophagosomes. Activation of the autophagic pathway by inhibition of the Shh pathway attenuates apoptosis by multiple pathways including the activation of NF-kB and JNK by TAK1. Autophagy itself appears to also suppress apoptosis, albeit its underlying mechanism remains unknown. In addition, it is also not clear how Gli1 suppression leads to TAK1 activation.

Finally, we investigated if inhibition of autophagy also enhanced GANT61-mediated antiproliferative activity. GANT61 incubated with SW1736 for 72 h (Fig. 6A) or 48 h (Fig. 6B) decreased SW1736 cell proliferation largely in a dose-dependent manner. CQ (10 μM) or 5Z (2 or 5 μM) alone inhibited SW1736 cell proliferation by ~50–60%. CQ (10 μM) or 5Z (2 or 5 μM) in combination with various concentrations of GANT61 had additive effects on cell proliferation, compared to those treated with CQ, 5Z, or GANT61 alone (Fig. 6A, B).

## Discussion

The Shh pathway is primarily recognized for its roles in tumor cell proliferation, drug resistance, metastasis, and stem cell self-renewal^5^. Several prior studies have shown that inhibition of this pathway induces autophagy in neuroblastomas and in breast, hepatocellular, and lung cancers^11,12,14,22^. However, the mechanisms of autophagy induction and its cytoprotective activity are poorly understood. Our present study provides evidence that inhibition of Gli1 and Smo activity by their specific inhibitors or by Gli1 siRNA induces autophagy through the activation of TAK1, a serine/threonine kinase that activates two downstream kinases, AMPK and JNK. AMPK activation leads to ULK1^S555^ phosphorylation and activation. Inhibition of JNK and AMPK activity by their specific inhibitors blocks GANT61-induced autophagy. We further show that blocking autophagy by chloroquine and 5Z and by TAK1 and Beclin-1 siRNA potentiates the apoptotic effect of GANT61 in SW1736 cells. Our study provides novel insights into the mechanisms by which inhibition of the Shh pathway induces autophagy.

TAK1 regulates a plethora of cellular functions including survival, apoptosis, differentiation, and the innate immune response^18^. TAK1 can be activated by various extracellular stimuli such as TGF-β, IL-1, TNF-α, LPS, DNA damage, microbial pathogens, and Toll-like receptors (TLR)^23^. TAK1 is a member of the MAP 3 kinase (MAP3K) family and activates multiple downstream MAP kinases including the JNK, ERK, and p38 pathways. TAK1 also activates IKKβ and its downstream NF-κB^24^. It has been increasingly appreciated that TAK1 can phosphorylate and activate AMPK to induce autophagy^17^. TAK1 is required for TGF-β−induced autophagy in murine mesangial cells through the TAK1-MKK3-p38 signaling pathway^25^ and is required for tumor necrosis factor-related apoptosis-inducing ligand (TRAIL)-induced autophagy in human epithelial cells^26^. TAK1 activates AMPK to induce autophagy in response to S6K1 suppression^27,28^. TAK1 plays an important role in LPS and microbial pathogen-induced autophagy^29,30^. Our present study provides several lines of evidence showing that TAK1 activation plays an important role in Shh pathway inhibition-induced autophagy in thyroid tumor cell lines: (1) The inhibitors of Gli1 and Smo as well as Gli1 siRNA induced TAK1 phosphorylation, LC3 lipidation, and the formation of autophagosomes; (2) Gli1 overexpression decreased the basal levels of LC3 lipidation; (3) TAK1 inhibition by 5Z and by siRNA blocked GANT61-induced autophagy and phosphorylation of AMPK and JNK. These observations collectively suggest that TAK1 activation plays a crucial role in mediating Gli1 inhibition-induced autophagy (Fig. 6C).

AMPK can be activated by elevated intracellular AMP levels^20^ or by several protein kinases such as calcium/calmodulin-dependent kinase kinase (CaMKK), liver kinase B1 (LKB1), and TAK1 (Klionsky et al.^10^). Interestingly, AMPK activation by metformin phosphorylates Gli1 and downregulates its expression by destabilizing the Gli1 protein^31–33^. AMPK phosphorylates ULK1 at multiple sites, including S317, S468, S555, or S777. ULK1 phosphorylation and activation lead to autophagy induction^10^. We recently reported that TAK1 activation by inhibition of S6K1 activity or by LPS and microbial pathogens leads to AMPK activation and autophagy^27–30,34^. Consistent with these observations, our present study demonstrates that inhibition of the Shh pathway by GANT61 or by Gli1 knockdown led to increased AMPK and ULK1 phosphorylation and activation, and that AMPK activation contributed to GANT61-induced autophagy in thyroid cancer cell lines. It appears that the Shh pathway and AMPK regulate each other in a forward-feedback loop (Fig. 6C).

JNK has been implicated in playing an important role in inducing autophagy under various settings. For example, nutrient deprivation activates JNK to phosphorylate Bcl-2 at multiple sites, including T69, S70, and S87 (Klionsky et al.^10^). Phosphorylated Bcl-2 dissociates from the Bcl-2-Beclin-1 complex and participates in the formation of the pre-initiation complex and induction of autophagy^10^. JNK activation is involved in autophagy induced by hypoxia and chemotherapy^35^. We reported earlier that JNK activation by TAK1 is also involved in S6K1 inhibition-induced autophagy^27^. RITA, a p53 activator, downregulates the Shh pathway in a p53-independent and reactive oxygen species-independent manner and activates JNK; JNK activation is required for RITA-induced autophagy^36^. Our present study shows that GANT61 induced JNK phosphorylation, and that inhibition of JNK activity by SP600125 partially blocked GANT61-induced autophagy. Mechanistic investigation revealed that JNK activation was mediated by its upstream TAK1 (Fig. 6C). Our study provides evidence suggesting that inhibition of the Shh pathway activates JNK1, which contributes to GANT61-induced autophagy.

In the past two decades, there have been extensive studies focusing on the role of autophagy in drug resistance to chemo- and target therapy^21^. For example, autophagy induced by the inhibitors of B-Raf kinase, EGFR, and other receptor tyrosine kinases renders tumor cells resistant to the induction of apoptosis and compromises their antitumor activity^37,38^. Autophagy is also involved in chemoresistance to many genotoxic anticancer drugs^39^. There has been great interest in combining an autophagy inhibitor plus an anticancer drug to achieve synergistic antitumor activity. Vismodegib, a Smo inhibitor, induces autophagy in two lung adenocarcinoma cell lines and has weak antiproliferative activity in vitro and antitumor activity in vivo^14^. Inhibition of autophagy by ATG5 or ATG7 siRNA or by chloroquine enhances the antitumor activity of vismodegib^14^. Inhibition of GANT61-induced autophagy enhances apoptosis in neuroblastoma^12,13^. Our present study shows that GANT61 induced autophagy and apoptosis in two thyroid tumor cell lines. Inhibition of autophagy by Beclin-1 or TAK1 knockdown or by chloroquine enhanced GANT61-induced apoptosis. These observations collectively suggest that autophagy compromises the antitumor activity of GANT61. In contrast, Wang et al.^11^ reported earlier that inhibition of autophagy by 3-methyadenine or Beclin-1 knockdown suppressed GANT61-induced apoptosis and cytotoxicity in hepatocellular carcinomas. It is not clear if these contradicting observations result from different types of cancer.

There are several unsolved issues in our current study. First, it is not clear how inhibition of the Shh pathway activated TAK1. TAK1 activation is complex and can be regulated by multiple mechanisms. TAK1 becomes activated when it interacts with TAK1 binding protein-1 (TAB1) plus TAB2 or TAB3, the latter is modified by K63-linked polyubiquitination following a variety of stimuli such as IL-1 and TNF-α^18^. O-linked GlcNAcylation of TAB1 by osmotic shock or IL-1 and phosphorylation by the calcium calmodulin-dependent kinase CaMKII also activate TAK1 (Mukhopadhyay et al.^18^) . TAK1 is also activated by TRAF6 through ataxia-telangiectasia mutated (ATM) in DNA-damaged tumor cells^40–42^. It is not clear if GANT61 activated TAK1 by activating ATM. Second, p62 is usually degraded in the autophagic process^10^. Our present study showed that GANT61 did not decrease but rather increased p62 levels in SW1726 and KAT-18 cells. Previous studies have shown that JNK activation induces p62 expression by autophagy inducers such as resveratrol or dehydroepiandrosterone^10,43,44^. We conducted RT-PCR and the p62 promoter-driven luciferase reporter assay and unexpectedly found that GANT61 did not increase p62 mRNA levels nor its promoter activity (data not shown). Kehl et al.^45^ recently reported that TAK1 can protect p62 from autophagy-mediated degradation by reducing p62 translocation into autophagosomes. Since TAK1 was readily activated by GANT61, we speculate that increased p62 levels in GANT61-treated cells are due to TAK1-mediated protection. Third, while inhibition of autophagy enhanced GANT61-induced apoptosis, chloroquine or 5Z only enhanced the antiproliferative activity of GANT61 modestly when they were used in combination with GANT61 (Fig. 6A, B). It is not clear if GANT61 in combination with chloroquine or 5Z could dramatically improve their antitumor activity in vivo in a mouse tumor xenograft model; Fourth, the mechanisms by which autophagy antagonizes GANT61-induced apoptosis remain to be investigated (Fig. 6C); Fifth, it is well known that NF-κB activation leads to Bcl-2 expression and confers resistance to apoptosis in tumor cells^24,46^. Since TAK1 activated by GANT61 also induced NF-κB activation (Fig. 2A), it is likely that 5Z potentiates GANT61-induced apoptosis in part by blocking NF-κB activation (Fig. 6C). Sixth, phosphorylation and activation of JNK leads to its dissociation with Bcl-2. Bcl-2 released from the JNK-Bcl-2 complex is translocated into the mitochondrial membrane to suppress apoptosis (Fig. 6C). Enhanced apoptosis by TAK1 inhibition may be contributed in part by blocking JNK phosphorylation (Fig. 6C). Thus, TAK1 activation may attenuate GANT61-induced apoptosis by multiple pathways (Fig. 6C).

In summary, our present study has shown that inhibition of the Shh pathway cross-activates the TAK1-AMPK pathway in two anaplastic thyroid cancer cell lines. TAK1 activation leads to the phosphorylation of its two downstream substrates, AMPK and JNK. Activation of AMPK and JNK contributes to GANT61-induced autophagy in thyroid tumor cell autophagy. Inhibition of autophagy enhances GANT61-induced apoptosis and antiproliferative activity. Our study suggests that inhibition of the Shh pathway induces autophagy by activating the TAK1-AMPK axis and provides new insights into the mechanisms of GANT61-induced autophagy (Fig. 6C).

## Materials and methods

### Reagents

Cyclopamine (CP) and Compound C (CC) were purchased from Selleck Chemicals LLC (Shanghai, China) and dissolved in dimethyl sulfoxide (DMSO). Bafilomycin, chloroquine, SP600125, and 5Z-7-oxozeaenol (5Z) were purchased from Sigma (St. Louis, MO). GANT61 was purchased from Medkoo Biosciences (Morrisville, NC) and dissolved in 100% ethanol. Antibodies against Gli1 (2643), ULK1 (8054S), ULK1^S555^ (5869S), AMPK (5831S), AMPK^T172^ (2535S), ACC (3662S), ACC^S79^ (3661S), Beclin-1^S93^ (14717S), Beclin-1(3495S), ATG5 (12994S), ATG7 (8558S), p65^S539^ (3033S), ERK^S202/204^ (3510S), ERK (4685S), p38^T180/182^ (9211S), p38 (8690S), TAK1 (5206S), TAK1^S412^ (9339S), JNK (9252S), JNK^T183/185^ (4668S), Beclin-1 (3495S), caspase-3 (9662S), cleaved caspase-3 (9664S), caspase-8 (4790S), cleaved caspase-8 (9694S), LC3 (3868S), p62 (5114S), and PARP (9532S) were purchased from Cell Signaling Technology, Inc. (Danvers, MA). Antibodies against p65 (sc-372), β-Actin (sc-47778), and Glyceraldehyde 3-phosphate dehydrogenase (GAPDH) was obtained from Santa Cruz Biotechnology Inc. (San Diego, CA). Gli1 (SR301820C) siRNA ON-TARGETplus SMARTpools were synthesized by Dharmacon and purchased from Fisher Scientific (Pittsburg, PA). Beclin-1 siRNA (6246S) and TAK1 siRNA (6317S) were purchased from Cell Signaling Technology, Inc. (Danvers, MA). Fluorescein isothiocyanate (FITC)-labeled Annexin V Apoptosis Detection Kit (556547) were acquired from BD Biosciences, Inc. (Shanghai, China). Green fluorescence protein (GFP)-microtubule-associated protein 1 light chain 3 (GFP-LC3) plasmid was kindly provided by Dr. Quan Zhang (Yangzhou University, Yangzhou, China).

### Cell lines and plasmid DNA

Two ATC cell lines, KAT-18 and SW1736, were kindly provided by Dr. Kenneth B. Ain, authenticated recently^9^. KAT-18 cells were used between 40 and 60 passages; SW1736 cells were used between 26 and 45 passages.

### Western blot

SW1736 and KAT-18 cells seeded in six-well plates were treated with the indicated concentrations of cyclopamine or GANT61 for 48 h or transfected with a Gli1 expression vector according to the manufacturer’s instructions. Cell lysates were prepared as described^47^ and analyzed for the expression of the indicated proteins. For loading controls, β-actin was detected by a mouse monoclonal antibody. Relative protein levels were analyzed by quantifying the blots with NIH Image-J software and presented as bar graphs.

### Gene knockdown

KAT-18 and SW1736 cells seeded in a six-well plate were transfected with Gli1 by using Lipofectamine RNAiMAX (Invitrogen Life Technologies, Grand Island, NY) according to the manufacturer’s instructions. A scrambled siRNA was included as a negative control. After incubation for 48 h, cell lysates were analyzed by Gli1, LC3, p62, and β-actin expression by western blot with their specific antibodies. To determine the effect of inhibition of autophagy on GANT61-induced apoptosis, SW1736 cells were transfected with TAK1 and Beclin-1 siRNA. A scrambled control siRNA was included as a negative control. After transfection for 48 h, the cells were left untreated or treated with GANT61 (10 μM) and then incubated for 24 h. Cell lysates were prepared and analyzed for TAK1, Beclin-1, caspase-8, caspase-3, PARP, and β-actin.

### GFP-LC3 fluorescence analysis

SW1736 cells seeded on coverslips were transiently transfected with GFP-LC3 expression plasmid DNA using FuGENE6 following the manufacturer’s protocol. After incubation for 48 h, the cells were incubated in the absence or presence of GANT61 (10 μM) minus or plus bafilomycin (10 nM) or chloroquine (10 μM; Fig. 1D), 5Z (1 μM; Fig. 3B), CC (5 μM; Fig. 4B), SP600125 (10 μM; Fig. 4B). After incubation for 48 h, the cells were fixed in 100% methanol at −20 °C for 10 min. The coverslips were mounted with 50% glycerin in PBS containing 4,6-diamidino-2-phenylindole (DAPI; 0.5 μg/ml; Sigma Chemical Co.). Autophagosomes were examined under a Leica LP8 confocal microscope. The autophagosome puncta was examined under a Nikon fluorescence microscope. Autophagosome puncta in SW1736 cells treated with various drugs were counted in 10 randomly selected fields under a ×40 objective in a blinded fashion. Results represent the mean ± SD (standard error of deviation) of three independent experiments.

### Apoptosis assay

SW1736 cells seeded in six-well plates were incubated in the absence or presence of GANT61 (10 μM) minus or plus 5Z (5 μM) (**A**) or CQ (10 μM) (**B**) for 24 h. Single-cell suspensions were prepared and stained for propidium iodide (PI) and annexin V by using a FITC Annexin V Apoptosis Detection kit following the manufacturer’s instructions. Single-cell suspensions were run in a Beckman Coulter flow cytometer (Model CyAn ADP). The fluorescence intensity was analyzed by using the FlowJo software. Annexin-positive cells were gated. The percentage of Annexin V-positive cells from three independent experiments were calculated and statistically analyzed by using the unpaired Student’s *t* test. Data in bar graphs are the mean ± SD of three independent experiments.

### Cell viability assay

SW1736 cells seeded in 96-well plates (2000 cells/well) were incubated with the indicated concentrations of GANT61 minus or plus CQ (10 μM; Fig. 6A) for 72 h or 5Z (2 or 5 μM; Fig. 6B) for 48 h. Cell viability was measured by using a CellTiter-Glo kit (Promega, Madison, WI, USA). Data are the mean ± SD of the triplicate from one representative of three independent experiments with similar results.

### Statistical analysis

The differences in the density of western blots, autophagosome puncta, cell viability, and apoptosis between different treatment groups were statistically analyzed by using an unpaired Student’s *t* test or Analysis of Variance (ANOVA) when the data set has three or more independent groups. The *p* value of <0.05 was considered statistically significant. All statistics was performed with SigmaPlot 11 software (Systat Software, Inc, San Jose, CA).
